# Shaping the future of spinal implants: advancing bioactive composites with 3D printing for next-generation surgical care

**DOI:** 10.1186/s13018-026-06772-w

**Published:** 2026-02-28

**Authors:** Naresh Kumar, Namith Rangaswamy, Si Jian Hui, Lionel Yan Jin Lee, Praveen Jeyachandran, Niyou Wang, Jerry Ying Hsi Fuh, A. Senthil Kumar, James Thomas Patrick Decourcy Hallinan, Balamurugan A. Vellayappan

**Affiliations:** 1 Department of Orthopaedic Surgery, National University Hospital, National University Health System Tower Block, Level 11, 1E Kent Ridge Road, Singapore, 119228 Singapore; 2 Department of Orthopaedic Surgery, National University Singapore, Level 11, Tower Block, 1E Kent Ridge Road, Singapore, 119228 Singapore; 3 Institute of Materials Research and Engineering, Agency for Science, Technology, and Research, 2 Fusionopolis Way, Innovis 08-03, Singapore, 138634 Singapore; 4 Department of Mechanical Engineering, College of Design and Engineering, National University of Singapore, 9 Engineering Drive 1, Singapore, 117575 Singapore; 5 Department of Diagnostic Imaging, National University Hospital, National University Hospital, Main Building, Level 2, 5 Lower Kent Ridge Road, Singapore, 119074 Singapore; 6 Department of Radiation Oncology, National University Health System, Level 7, Tower Block, 1E Kent Ridge Road, Singapore, 119228 Singapore

**Keywords:** Spine implants, PEEK, Metastatic spine disease, Osteoporosis, Additive manufacturing

## Abstract

The evolution of spinal implant materials must keep pace with advances in our understanding of bone biomechanics and changing clinical demands in spine surgery. While early innovations primarily addressed trauma and spinal deformities using titanium alloys as the gold standard, an aging population has led to an increasing detection of osteoporosis and metastatic spine disease. These conditions fundamentally alter bone quality and mechanical behaviour and pose significant challenges for traditional metallic implants. These are not only due to stress shielding and increased risk of failure in compromised bone but also their radiopacity, which produces imaging artifacts that obscure anatomical detail and complicate disease monitoring and radiotherapy. Innovations in advanced polymers, bioactive material integrations, and additive manufacturing have delivered a glimpse into the next generation of implants tailored to the multifaceted demands of modern spinal surgery. This paper studies the evolution of spinal pathologies to deliver the guided engineering of future implant materials that offer optimal mechanical compatibility, biological integrability, radiological advantages, and allow for enhanced processibility and customization for patient-specific applications. This paradigm shift promises improved implant longevity, enhanced biological response, superior imaging and radiotherapy outcomes, and greater potential for personalized solutions to ultimately advance care for patients with osteoporosis, metastatic disease, and other complex spinal conditions.

## Introduction

Orthopaedic and spinal surgery have undergone significant progress over the last century, driven by changes in spinal disorder management and the progress in metallurgical sciences. The development of implantable hardware has transformed spinal implants from early rudimentary fixation devices to modern biomechanically sophisticated implant systems. These systems are designed to provide stability, restore spinal alignment, and promote fusion when necessary.

Historically, implants primarily managed trauma and deformities. The Edwin Smith Papyrus (thirtieth century BC) is among the earliest available record detailing the use of natural materials to treat orthopaedic pathologies [1]. Modernization and the invention of X-rays by Wilhelm Roentgen accelerated the understanding of skeletal anatomy and fostered implant-based management strategies for spinal pathologies [2]. This led to advanced surgical techniques like the first use of wires to wrap the spinal column of a fractured spine by Dr Berthold Hadra in the nineteenth century [3], and bolder approaches like the use of steel bars and silk wires to treat scoliosis by Dr. Paul Harrington in the mid-twentieth century [4].

The increasing life expectancy of the general population is driving the rise in spine surgeries with osteoporotic and metastatic spine tumour surgeries comprising nearly half of all procedures [5]. This demand places a substantial burden on healthcare systems worldwide and urgently calls for a new era of biomaterials with unique properties, especially in metastatic cases where implants should minimally interfere with image-guided radiotherapy and postoperative follow-up imaging [6]. Therefore, healthcare technology and surgical techniques must evolve symbiotically. Implant materials must now extend beyond mechanical properties close to bone to reduce stress shielding, and biocompatibility to prevent implant loosening. While titanium alloys remain the gold standard for trauma and deformity management [7], a re-evaluation of this standard is necessary to address the evolving demands of modern spinal surgery. Surgeons now seek robust implants with enhanced bio-functionality, biocompatibility, and biostability, driving the ongoing development of novel implant materials for a modernized society.

This study reviews the evolution of spinal biomaterials used for fixation and provides a perspective on the desirable qualities of emerging biomaterials to manage osteoporosis and metastatic disease. It will also address the strategic deployment of advanced engineering technologies for accelerated research and development of biomaterials to meet contemporary clinical needs.

## Yesterday’s ‘gold standard’

### Biomechanical property of bone guiding implant material developments

Bone is the principal weight-bearing structure of the human body, uniquely composed of cellular material, collagen fibres, and mineralized inorganic matrix arranged in a distinct microarchitecture. Based on density and structural arrangement, bone is classified into dense cortical or spongy cancellous. This composite structure gives bone its anisotropic properties with varying biomechanical characteristics according to load direction and magnitude [8]. For instance, cortical bone with parallel osteons offers greater stiffness and resistance to deformation but fails at lower strains (~ 2%). In contrast, trabecular bone withstands much higher strains of up to 30% due to its porous architecture [9, 10].

A key parameter guiding implant design is apparent density, which is defined as the product of volume fraction and material density of trabeculae. The density shows a power-law relationship with the elastic modulus of bone [11], which is a measure of resistance to deformation [12] and varies between 1.4–17 GPa [13–18]. Matching an implant’s modulus to that of native bone is essential for effective stress transfer and to avoid interfacial failure, particularly in spinal surgery where implants must withstand substantial compressive and torsional forces.

**Fig. 1:**
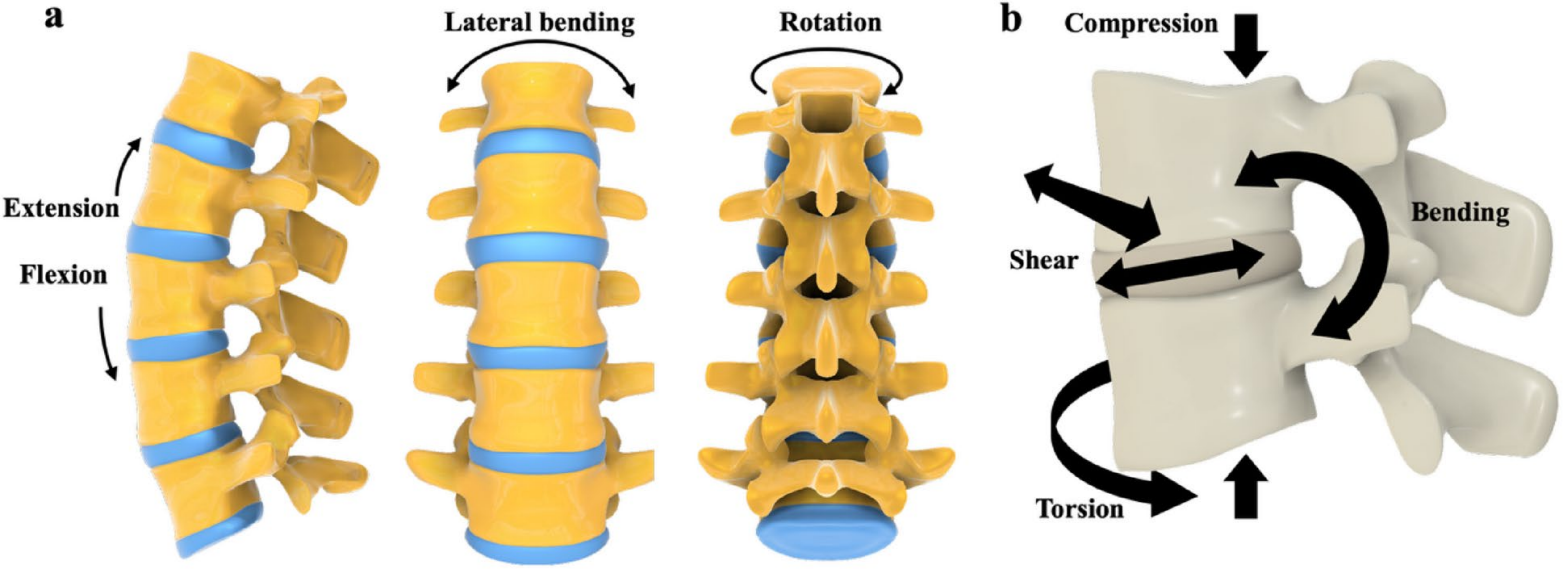
Bone biomechanics: **a** Key degrees of motion along the spine; **b** physiological loading conditions along the spine

Most of these observations were derived from linear loading studies, but a multi-axial failure criterion has yet to be universally established [19]. Biomechanical analyses illustrate the complexity of physiological loading conditions along the spinal column as illustrated in Fig. 1. Activities involving lateral bending, deep flexion, and large trunk rotations generate significant shear and strain across the vertebral column, with failure typically occurring through lateral bending and endplate separation [20]. Finite element analysis estimates that the anterior spinal column can experience compressive forces of 400–1,200 N and shear forces of 150–500 N [21]. Therefore, the engineering of suitable implant materials for surgical immobilization or fusion becomes critical in meeting both the physiological loading conditions and the disruptions in natural force transfer through the spinal column. These necessities have led to the widespread adoption of metallic biomaterials in orthopaedic and spinal applications that are valued for their mechanical strength and durability.

### Metallic implant materials: titanium alloys

Stainless steel was initially employed for spinal implants due to its ease of fabrication, availability, and perceived biocompatibility [22]. However, its high modulus of elasticity (193 GPa) created a significant mismatch with bone, leading to ‘stress shielding’ where the stiffer implant absorbs most of the mechanical load. This effect eventually caused localized bone density loss and implant loosening over time [23]. Additional drawbacks included corrosion, metal ion release, adverse local tissue reactions, and interference with imaging due to its ferromagnetic properties [24–26].

The introduction of Ti6Al4V in the late twentieth century marked a major advancement in implant technology paving the way for the current gold standard for spinal and orthopaedic implants. The medical-grade titanium alloy offers lower modulus of elasticity (110 GPa), lighter weight, enhanced corrosion resistance [27], and superior biocompatibility when compared to stainless steel [28]. Furthermore, Ti6Al4V promotes osteointegration especially with surface modifications that facilitate bone cell attachment and proliferation [29, 30].

Titanium-based implants, including screws, rods, cages, and plates, have proven effective in treating spinal trauma, deformities, and degenerative diseases by providing stable fixation [31]. Despite these advances, Ti6Al4V is not without limitations. Its modulus of elasticity remains substantially higher than that of both healthy and diseased bone, raising persistent concerns about stress shielding especially in osteoporosis and metastatic cancer. Nonetheless, the evolution of Ti6Al4V has underscore critical lessons in biomaterial development, including the importance of mechanical compatibility, corrosion resistance, and osteointegration.

## Evolving spinal healthcare needs

The lessons learnt from Ti6Al4V provide the foundation for the innovation of next-generation biomaterials tailored to the unique challenges of diseased bone. To inform the design of such materials, it is essential to first characterize how conditions like osteoporosis and metastasis specifically alter bone composition, architecture, and mechanical behaviour. These pathological changes will set the context for the development of advanced biomaterials that can better meet the needs of patients with compromised bone quality.

### Change in mechanical properties of bone in osteoporosis and metastasis

**Fig. 2:**
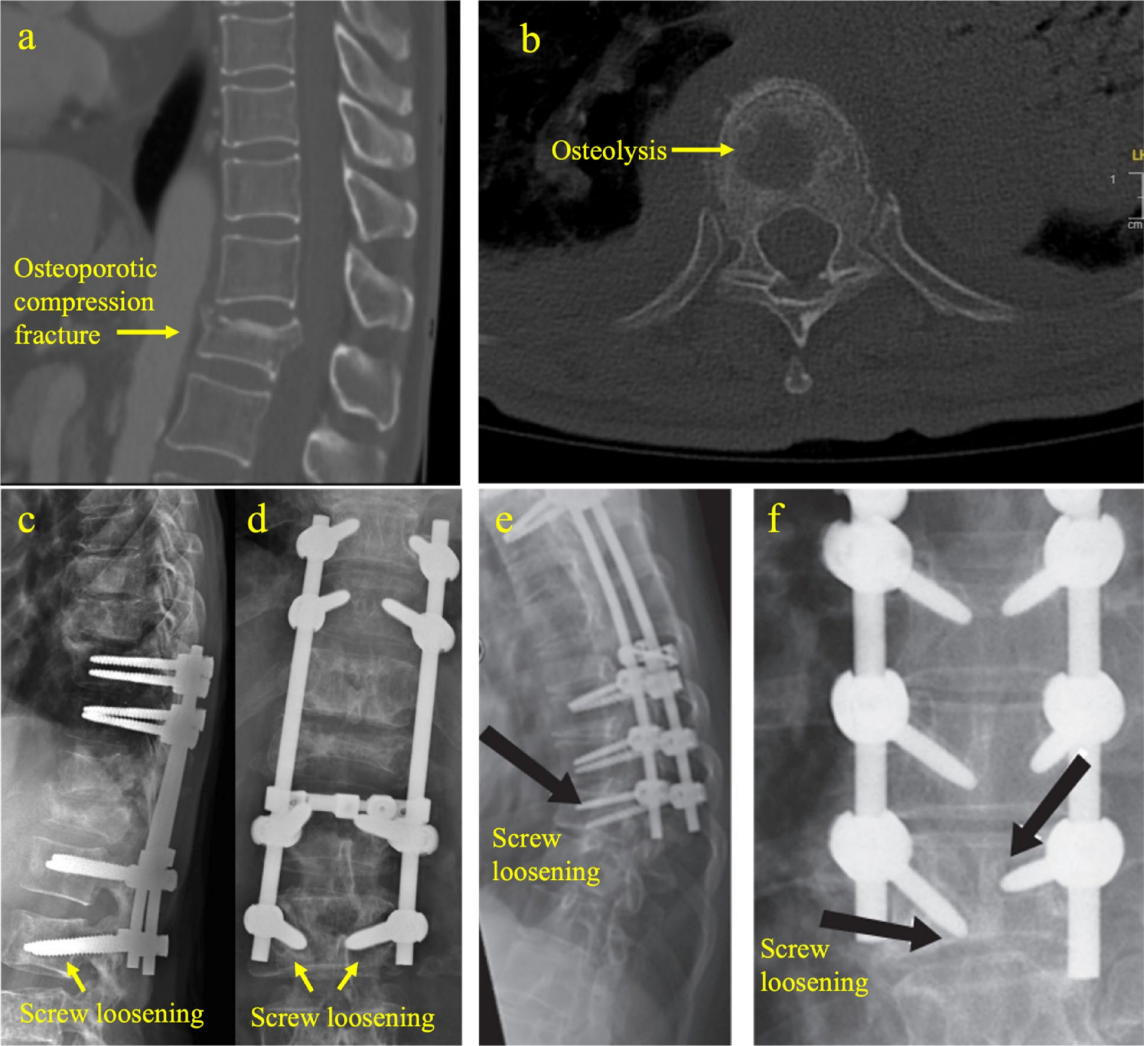
**a** Osteoporotic compression fracture. **b** Osteolytic metastatic lesions in the thoracic spine. **c**, **d** Screw loosening at L3 in a cancer patient. **e**, **f** Postoperative screw loosening at T8 and T9 at 4 months in 66-year-old male with chronic myeloid leukaemia [53]

Bone adapts to changing loads by modifying its structural density and orienting its microarchitecture along loading directions. This adaptive capacity relies on continuous remodelling, balancing bone resorption and formation. With ageing, bone remodelling activity becomes unbalanced, favouring resorption and negatively resulting in osteoporosis [32]. Early osteoporosis primarily affects trabecular bone before progressing to cortical bone loss in advanced stages [33]. Epidemiological data reflects this phenomenon by vertebral compression fractures in trabecular bone occurring before age 65 (Fig. 2a), while cortical hip fractures are more common in older individuals [34]. Meanwhile, anterior wedge fractures in the vertebrae are linked to the region’s high anisotropy and low density [35–37]. The decline in bone strength and increase in fracture risk are results of the combined decrease in mineralization and disorganized microarchitecture [38, 39].

Moreover, studies show that the decrease in mineralization of osteoporotic bone with older individuals implies a corresponding loss in Young’s modulus [40, 41]. Osteoporotic bone typically corresponds with bone density and has been consistently modelled by the reduction in elastic moduli. This is regardless of segregating cortical and cancellous bones [42, 43] or considering the loss in apparent moduli as a function of bone mineral density [44, 45]. The precise definition for the elastic modulus of osteoporotic bone is a complex problem that has yet to be determined, but the loss in elastic modulus of osteoporotic bone is an established consensus.

On the cancerous involvement of bone, particularly metastasis, direct biomechanical studies are limited [46] while radiographic and histological analysis serve as insightful surrogates. Metastatic lesions are classified as osteolytic, osteoblastic, or mixed. Osteoblastic lesions have increased bone-trabecular volume fraction (BV/TV) with thickened and shortened trabeculae [47–49], while osteolytic metastases show significant trabeculae thinning and volume losses as depicted in Fig. 2b [50]. Hence, both types compromise structural integrity of bone with projected lowering of fatigue thresholds and failure resistances.

### Failure of implants in osteoporosis and metastatic spinal disease

Implant failure is characterized by radiologically apparent disassembly or breakage without changes in adjacent bone, while ‘construct failure’ constitutes cases where surrounding bone is affected. Repeated loading along the spinal construct induces fatigue to implant junctions, such as screw heads and rods, often leading to breakage. In metastatic spine disease, destructive lytic bone lesions further undermine the mechanical capacity of bone to support traditional fixation techniques [51]. Studies, including our own, have detailed various failure modes in metastatic spinal disease, which can be clinically classified as symptomatic (with pain, irritation, or neurological symptoms) or asymptomatic (by radiological detection) [52, 53]. Symptomatic failure occurs in 5.7% of cases while asymptomatic failure is much higher at 41%. We and other contemporary researchers have observed that there is a rising incidence of bone-implant interfacial construct failure as depicted by halo observations around pedicle screws in Fig. 2c–f. This is in addition to the implant–implant junctional failure, which highlights the importance of bio-integration.

Similarly, in osteoporosis, reduced bone mineral density and altered microarchitecture can compromise the interface between the implant and the surrounding tissue, increasing the risk of loosening and mechanical failure [54]. Screw pull-outs, plough-outs, cut-throughs, and cage subsidence are expected to occur from poor osseous integration in weakened bone. Therefore, enhancing the bioactivity and bio-integration of implant materials is crucial to reduce future implant and construct failures, especially in patients with compromised bone quality.

## The search for the right implant in modern spine surgery

Despite titanium alloys being described as the “gold standard” for use in spine surgery, implant-related complications persist, such as screw loosening, cage subsidence, and rod fractures. Additionally, artifacts generated from titanium’s radiopacity obscure critical anatomic or tumour details in computed tomography (CT) and magnetic resonance imaging (MRI) scans. Poor imaging results consequently complicate postoperative follow-up and radiotherapy planning. This drives the search for novel spinal implant compositions that can establish the “gold standard” of the future.

**Fig. 3:**
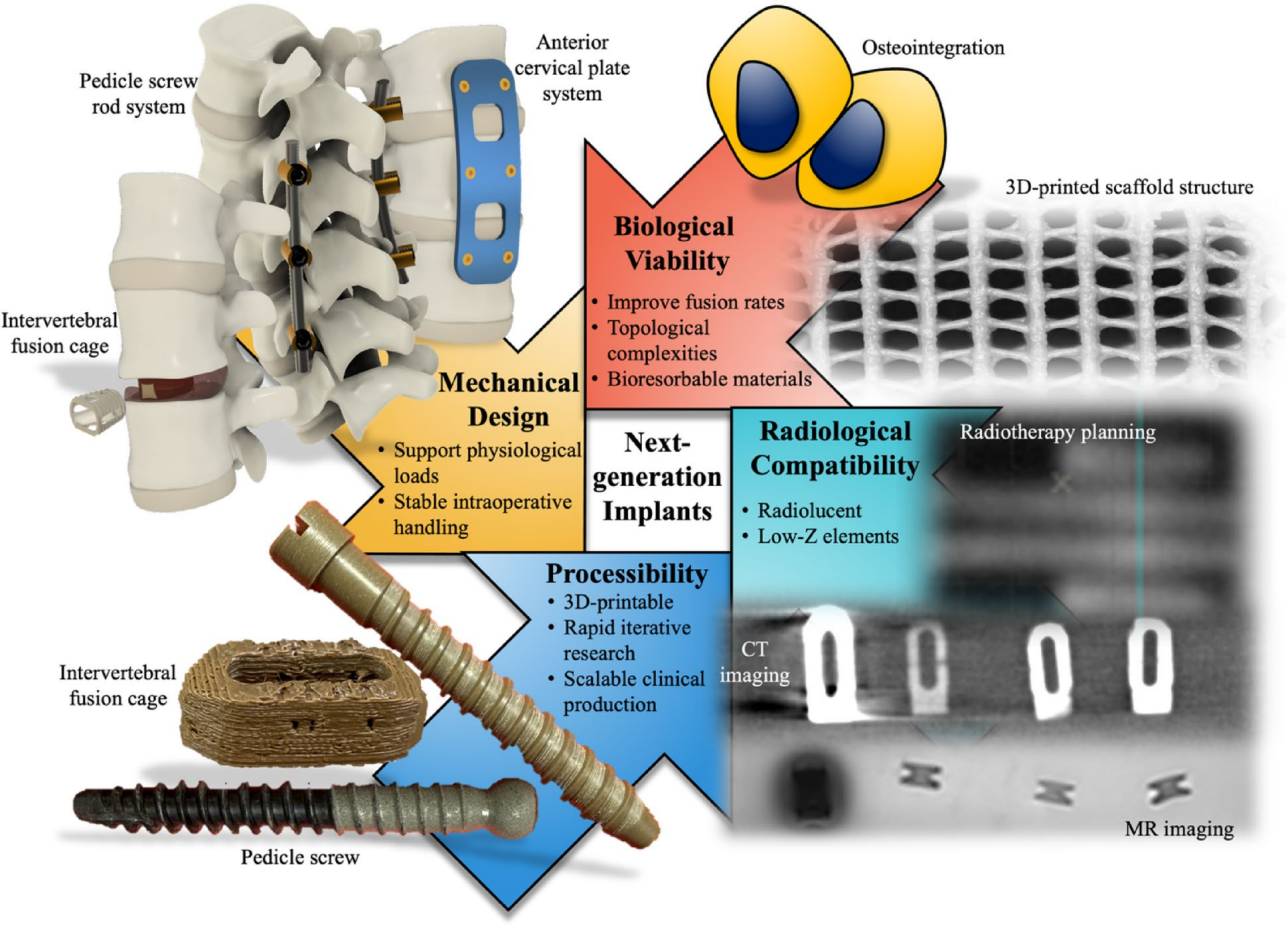
Critical considerations for next-generation implants encompassing mechanical design, biological viability, radiological compatibility, and processibility

Ultimately, the integration of advanced material science with pragmatic clinical needs is poised to transform spinal implant technology. This includes enabling on-demand production of patient-tailored solutions for a broader spectrum of spinal disorders. To this end, we must revisit the mechanical, biological, radiological, and processible qualities illustrated in Fig. 3 that play pivotal roles in guiding future spinal implant material designs to manage new-age osteoporotic and metastatic cases.

### Properties of next-generation spinal implants

#### Mechanical design

The primary objective of spinal implants remains to deliver structural stability and maintain spinal alignment. These are achieved with devices, such as the posterior pedicle screw and rod system for thoracic and lumbar procedures, anterior cervical plate systems incorporating plates and screws for cervical stabilization, and intervertebral fusion cages designed for fusion and restoration of disc height. Evidently, the diverse instrumentation used in spinal surgery necessitate implants with varying mechanical properties. For instance, pedicle screws and fixation rods require higher strength to resist permanent deformation under physiological loads thereby preserving structural integrity and alignment. These mechanical properties are also essential for stable intraoperative handling and manipulation, such as during contouring of fixation rods to match the patient’s spinal curvature. Conversely, fusion cages and plates are often engineered to mimic the mechanical properties of native bone while still providing sufficient compressive strength to support physiological loading. Meanwhile, it is critical to avoid excessively high mechanical properties to prevent stress shielding and its associated negative effects on bone remodelling. It should also be noted that there are spinal implants designed for intervertebral disc repair and replacement [55] but these aspects will not be covered in this paper, which focuses on surgical fixation implants with properties designed to tackle the increasing cases of osteoporosis and metastatic disease.

Biodegradable implants should satisfy the immediate physiological loading conditions upon implantation. However, the dissolution rate of implant materials should match osteogenesis rates, which can massively vary between patients particularly those with degenerative diseases. In the management of degenerative and oncological spine diseases, modification of implant geometry and using augmentations like cement are more commonly adopted strategies [56]. These approaches may include unique implant designs that reduce point loading [57] to minimize the risk of intervertebral cage subsidence and to accommodate spinal motion [58], or specialized pedicle screw thread designs to optimize fixation in osteoporotic bone [59]. Adjusting implant mechanical properties, such as reducing the elastic modulus of screw-rod systems, may help mitigate stress shielding for adjacent segment degeneration, though its effectiveness in osteoporosis remains unclear [60]. Ultimately, both mechanical properties and geometry must be strategically considered to optimize outcomes across different spinal pathologies.

#### Biological viability

Biocompatibility is a fundamental requirement for spinal implants to minimize local adverse reactions, such as inflammation or immune rejection. More importantly, bioactive materials are preferred for osteoporosis and metastatic spine disease where bone quality and healing are compromised. These materials can actively promote osteogenesis, accelerate bone-implant integration, and enhance structural stability for effective load transfer along the spinal column. Improved fusion rates and quality are particularly beneficial as robust bone-implant integration is typically delayed by poor osteoblastic activity of the osteoporotic condition. This leads to heavy reliance on instrumentation for anchorage within the host bone and temporary stabilization [61]. Additionally, insufficient bone-implant integration often leads to other mechanically unstable occurrences like micro-motion [62], implant loosening [63], and even debris formation [64].

Future directions in spinal implants for osteoporosis and metastatic spine disease are increasingly focused on achieving superior integration with host bone. Innovative surface modifications that increase topographical complexity can facilitate the attachment, proliferation, and differentiation of osteoblasts to accelerate new bone formation [65]. Meanwhile, scaffold-like implants that closely mimic the structure of the native bone extracellular matrix can provide an optimal environment for cellular infiltration and bone ingrowth [66]. Such scaffolds may be engineered from advanced biomaterials, including bioresorbable materials functionalized for the controlled release of therapeutic agents and biologically active agents like bone morphogenic proteins to stimulate local osteogenesis and enhance the regenerative capacity of compromised bone [67]. The convergence of material science, surface engineering, and molecular biology is expected to yield implants tailored to the challenges of poor bone quality and pathological bone loss, thereby improving clinical outcomes and patient quality of life.

#### Radiological compatibility

**Fig. 4:**
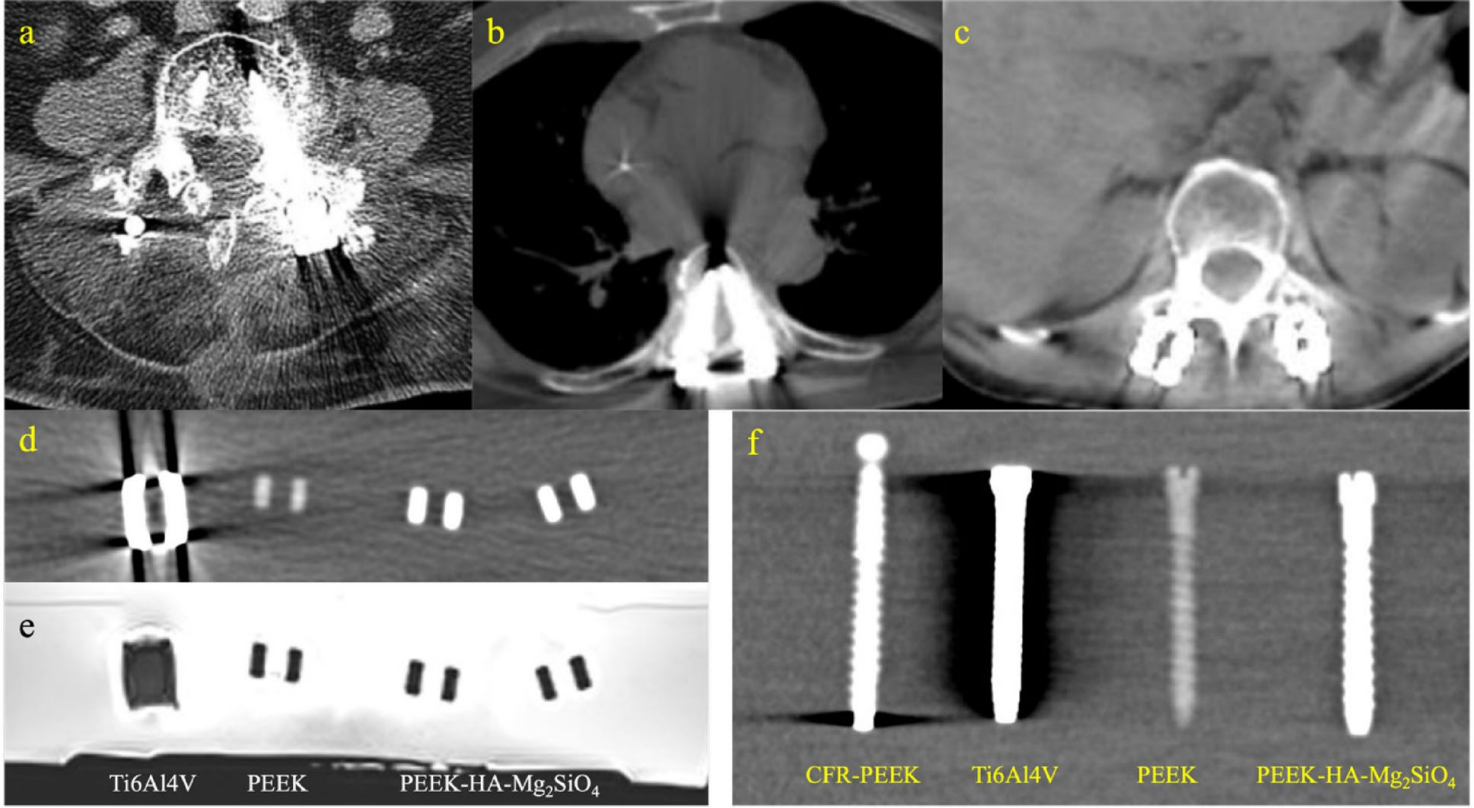
Implications of artifacts generated from implant biomaterials in CT and MR imaging: **a** Severe artifact generation around stainless-steel screws; **b** beam hardening artifacts in CT imaging at the tip of Ti6Al4V screws; **c** artifact reduction in CT imaging of CFR-PEEK screws; **d**–**e** CT and MR imaging comparison between Ti6Al4V, PEEK, and PEEK-HA- Mg_2_SiO_4_ cages showing severe artifacts around the Ti6Al4V cage, low CT image clarity of PEEK, and minimal artifacts around PEEK-HA-Mg_2_SiO_4_; **f** CT imaging comparison of artifacts generated around CFR-PEEK, Ti6Al4V, PEEK, and PEEK-HA-Mg_2_SiO_4_ pedicle screws

Radiological properties have become a key consideration in spinal implant selection, especially for patients with metastatic spine diseases. Postoperative imaging is vital for monitoring tumour recurrence and guiding precision radiotherapy, making the compatibility of implant materials with imaging and radiotherapeutic modalities crucial for optimal clinical outcomes. Artifacts compromise diagnostic quality and complicate follow-up assessments as shown by the severe artifacts in Fig. 4a–c that majorly hinder bone and implant identification.

Radiography serves both diagnostic and therapeutic roles in oncological spine care. High-resolution CT and MR imaging are essential for assessing the bone-implant interface, evaluating bone fusion, and detecting tumour progression or recurrence [68]. Meanwhile, advanced radiotherapy modalities, such as intensity-modulated radiotherapy (IMRT) and stereotactic body radiation therapy (SBRT), and precision photon and proton beam techniques require accurate knowledge of implant composition to ensure correct dose calculation and delivery. The interaction between implant and electromagnetic or particle beams can significantly impact both diagnostic imaging quality and radiotherapy accuracy [69].

Radiolucency is seen as a key property for minimizing imaging artifacts like beam hardening, photon scattering, and signal voids, which can obscure bone-implant interfaces or mask tumour recurrence on CT and MR imaging [70]. Meanwhile, excessive radiolucency may also affect implant visibility as depicted by PEEK in Fig. 4d, f. In radiotherapy, implants with high atomic number elements, such as titanium, can cause significant dose perturbation leading to discrepancies between planned and delivered doses and raising concerns over tumour control and the safety of nearby organs-at-risk [71]. Therefore, next-generation spinal implants are increasingly being developed with radiolucent and low-Z materials for oncological applications. Prioritizing a balance in these properties will improve both high-fidelity postoperative imaging and predictable radiotherapy precision to support diagnostic accuracy, therapeutic efficacy, and patient outcomes.

#### Material processibility

Considerable attention has been devoted to optimizing the mechanical and biological characteristics of implant materials. However, material processibility is an equally vital yet often overlooked factor. Processibility encompasses the ability to move from sustainable small-batch prototyping to scalable clinical manufacturing with the capacity to embrace patient-centric implant designs as part of a futuristic concept of personalized surgical planning. The advent of 3D printing has positioned processibility as another critical pillar in advancing next-generation spinal implants.

In the research of advanced biomaterial formulations, test materials are reliant on traditional manufacturing techniques, such as casting, moulding, and machining, which limits their capacity for rapid composition iteration. As clinical demands evolve and material composition complexities increase, these conventional approaches have become a bottleneck toward innovation and validation. Conversely, 3D printing offers rapid fabrication of test specimens and even near-net-shape prototypes [72] that accelerate iterative testing and optimization of novel implant material combinations and designs.

Additive manufacturing covers a wide range of metals, polymers, ceramics, and composites, through a variety of technologies like fused deposition modelling (FDM), stereolithography (SLA), and selective laser sintering (SLS). This versatility supports the fine-tuning of material properties to address specific clinical needs. It also makes it possible to produce complex geometries and porous architectures previously unattainable with conventional methods. The complexity of producible parts extends to the creation of patient-specific implants [72] by customizing implant geometry from anatomical imaging data. Clinicians can then achieve better fit, fixation, and adaptation to local bone quality or pathology, which are crucial for conditions like osteoporosis and metastatic spine disease. This patient-centric approach is expected to improve clinical outcomes by enhancing implant performance, minimizing intraoperative adjustments, and potentially reducing complications rates.

Despite these benefits, routine clinical integration of 3D printing into pre-operative surgical planning remains an envisioned concept with ongoing challenges in regulatory hurdles, validation of new material-process pairs, streamlining digital workflows to translate imaging data into manufacturable implants, and capabilities for swift on-demand operable implant delivery during pre-operative phases. Nonetheless, the synergy of additive manufacturing, artificial intelligence-driven design, and advanced biomaterials is poised to overcome these obstacles [73]. Therefore, material processibility is foundational to next-generation spinal implant innovation, especially when coupled with the versatile rapid prototyping capabilities of 3D printing that enable accelerated material innovation, scalable clinical production, and the realization of personalized spinal care.

### Next-generation biomaterials for osteoporosis and metastasis

#### PEEK as a reliable biomaterial

Over the past three decades, there has been a significant drive to identify next-generation biomaterials for spinal implants, with polyetheretherketone (PEEK) as a leading candidate. PEEK’s prominence is primarily attributed to its elastic modulus (3.5 GPa), which is much closer to that of cortical bone (12 GPa) than traditional metallic implants [63]. Additionally, its radiolucency allows for significantly clearer postoperative imaging. The clinical adoption of PEEK for spinal fusion cages began in the 1990s, as it addressed critical limitations of titanium implants, notably the risk of stress shielding and poor visualization of bone healing on radiographs. PEEK’s lower modulus of elasticity also promotes more physiological load sharing between implant and vertebrae, which in turn facilitates earlier and more reliable bony fusion.

Despite these advantages, PEEK has seen an overall decline in utilization rate over the last few years with metals reemerging as the preferred choice for osteoinduction. By 2022, 21% of interbody fusion procedures in the United States used PEEK compared to 65% that used metals like titanium and trabecular metal [74]. This was despite PEEK cages being the preferred choice for selected conditions, such as 68.1% of patients with degenerate disc disease receiving PEEK cages from 2007 to 2022. However, other clinical entities like spinal stenosis were treated with titanium cages accounting for their use in 82.3% of patients [75]. The bioinert nature of PEEK does not intrinsically support bone on-growth and impedes optimal fusion rates, which highlights the major limitation of PEEK as the optimal material for spinal surgery. Additionally, PEEK’s mechanical strength, particularly under cyclic shear and fatigue loads, is lower than that of metals, restricting its use in applications such as pedicle screws and rods that experience significant mechanical stress throughout the postoperative period.

#### Emergence of PEEK composites

To overcome the mechanical limitations of pure PEEK, carbon fibre reinforced PEEK (CFR-PEEK) stands out for its enhanced mechanical properties (18 GPa) and fatigue resistance. Clinically applied CFR-PEEK rods and plates have shown promise in both degenerative and oncologic spine applications. This is largely due to their lower radiographic artifact generation, which allows for more precise imaging of vertebral structures and lesions [6]. However, similar to unmodified PEEK, CFR-PEEK remains fundamentally bioinert, and its biological limitations underscore the ongoing need for further innovations in spinal implant materials.

Beyond CFR-PEEK, researchers have investigated composites and surface modification strategies. These include incorporation of rough or porous surface topographies [76] and blending PEEK with bioactive compounds to enhance osteointegration and mechanical performance [77], such as hydroxyapatite [78] calcium silicate [79], and titanium dioxide [80]. Early clinical studies have shown that some modified PEEK implants can achieve fusion rates comparable to titanium cages, while preserving the imaging advantages of radiolucency [81]. Contrarily, other theoretically ideal techniques like titanium-coated PEEK have shown debatable impact on clinical outcomes [82] with challenges like the introduction of debris during impaction [83].

Much of the literature has only partially addressed the broader criteria essential for advancing next-generation biomaterials. Further in-depth assessment is necessary for novel biomaterials with radiological compatibility, scalability of material processing (including rapid prototyping), and alignment with futuristic patient-centric treatment paradigms. In this context, our recent development of a 3D printable PEEK-HA-Mg_2_SiO_4_ composite [84] stands as a prime example of the necessary shift in research scope and methodology, specifically targeting the multifaceted requirements of future spinal implants.

#### Embracing additive manufacturing technology

The research on PEEK-HA-Mg_2_SiO_4_ highlights the comprehensive approach to exploring next-generation spinal implants while critically considering manufacturing complexities. Discussions on the composite itself are far less important than the conceptualized methodologies in developing an implant material that address the requirements of spinal implants. By integrating bioactive fillers like hydroxyapatite (HA) and magnesium orthosilicate (Mg_2_SiO_4_) into the PEEK matrix, a composite can be designed to achieve the multifaceted requirements of an advanced biomaterial for spinal implants. This included optimization of both biological [85] and mechanical [86] functions while also considering radiological compatibility.

More importantly, the processibility of PEEK-HA-Mg_2_SiO_4_ allowed the use of additive manufacturing technology for a systematic study on the progression of filler inclusion (e.g., PEEK-HA followed by PEEK-HA-Mg_2_SiO_4_) and filler concentration. This was achieved by varying the volumetric fraction of raw material powder being fed into a pellet-based 3D-printing system. Easy material composition iterations with 3D-printing may therefore accelerate biomaterial innovation and development to determine compositions capable of balancing the multifaceted requirements of spinal implants [86].

**Fig. 5:**
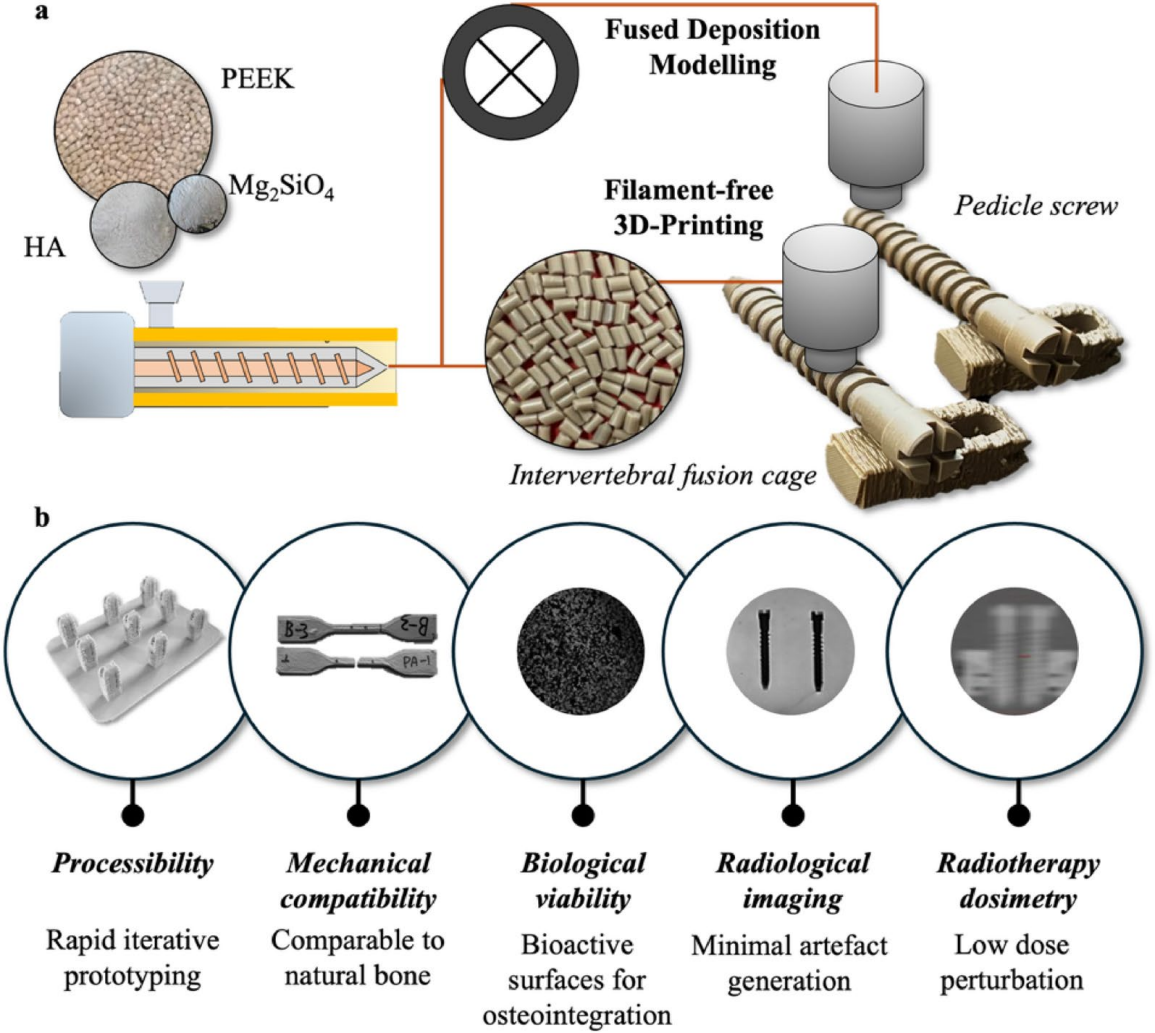
**a** Accelerating iterative implant material development with additive manufacturing technologies for PEEK-HA-Mg_2_SiO_4_ composites into implant structures; **b** Target properties of next-generation implants for spinal diseases in a modernized society

The integration of these bioactive particles directly into the bulk polymer matrix, particularly through modern filament-free additive manufacturing, offers advantages over surface coatings, such as reduced risk of delamination or uneven wear.^87^ Moreover, additive manufacturing facilitated the iteration of multi-materials and compositions in complex architectures. With 3D-printing, rapid prototyping of implant structures like intervertebral cages and pedicle screws (Fig. 5a) could be easily fabricated to international test standards (ASTM F2077, F2267, F543, etc.) for evaluation toward obtaining regulatory approval.

Our work exemplifies the ongoing evolution from standardized off-the-shelf implants to dynamic and bespoke devices engineered for seamless bone-implant integration in spine surgery. By systematically addressing the mechanical, biological, radiological, and processibility criteria (Fig. 5b), our research methodology demonstrates a substantial step toward innovation for next-generation biomaterials.

The outlined perspective for next-generation implant material development highlights 3D-printing as the advanced technology that can accelerate parameter iteration during research. Moving forward, a four-dimensional framework is proposed in Table 1 to guide future research in spinal implant development with the incorporation of 3D-printing technology for accelerated material and design innovation. As we continue to refine the adoption of additive manufacturing into biomaterial development, we anticipate even greater fidelity in replicating complex structures, improved scalability, and more consistent bioactive phase integration.

**Table 1:** Future directions for research in spinal implants enabled by 3D-printing

**Criteria**	**Future directions enabled by 3D-printing**
Mechanical	Fatigue, wear, and micromotion resistance under physiological loading Implant geometry and topology optimization with patient-specific computational modelling Tuneable composite architecture with graded stiffness to balance load sharing, minimize stress shielding, and resist fatigue Digital twins for preoperative design validation and postoperative performance prediction
Biological	Advanced surface engineering with nano/micro-topographies Scaffold-like porous architectures mimicking extracellular matrix to support osteogenesis Multi-material libraries and predictive models for patient-specific composite formulations to forecast osteoinductive potential and fusion likelihood in osteoporotic/metastatic bone
Radiological	Low-Z radiolucent composite formulations to reduce CT/MR artifacts while preserving mechanical performance Integration of implant geometry and material distribution to minimize scattering and beam hardening
Processibility and clinical translation	Cost-effective high-throughput formulation and screening for polymer/composite formulation iteration during research Qualification of additive manufacturing workflows with real-world performance Artificial intelligence-driven analytics to iterate designs and refine patient-selection criteria

## Conclusion

The evolution of spinal implant biomaterials has been guided by an ever-deepening understanding of spinal biomechanics, disease-specific challenges, and the clinical demands of modern spine surgery. While titanium alloys have been regarded as the “gold standard”, their biomechanical limitations have highlighted the need for further innovation, particularly in the context of complex pathologies like osteoporosis and metastatic spinal disease management. The lessons learnt from these metallic implants now inform the design of next-generation biomaterials that aim to overcome traditional barriers, such as stress shielding and imaging artifacts.

Looking ahead, the convergence of material science, molecular biology, and particularly additive manufacturing is paving the way for the development of advanced composite biomaterials, that offer tailored mechanical properties, enhanced bioactivity, radiological compatibility, and scalable processibility. The incorporation of additive manufacturing particularly enables fast iterations in biomaterial developments and marks a paradigm shift toward patient-tailored and disease-specific implants capable of addressing the multifaceted needs of an aging and increasingly complex patient population. Continued interdisciplinary research and clinical validation will be essential in translating these technological advances into improved outcomes and quality of life for patients undergoing spinal surgery.

## Data Availability

The dataset supporting the conclusions of this article is included within the article and available upon request to the authors.
